# Editorial: Role of toxicants, pollutants, and trace elements in health and nutrition

**DOI:** 10.3389/fnut.2023.1142959

**Published:** 2023-01-26

**Authors:** Abu Mohd Naser, Syed Moshfiqur Rahman, Maria Kippler

**Affiliations:** ^1^Division of Epidemiology, Biostatistics, and Environmental Health, School of Public Health, University of Memphis, Memphis, TN, United States; ^2^Department of Women's and Children's Health, Uppsala University, Uppsala, Sweden; ^3^Unit of Metals and Health, Institute of Environmental Medicine, Karolinska Institutet, Stockholm, Sweden

**Keywords:** environmental pollutants, toxicants, nutrients, trace elements, metals, acrylamide, aflatoxin B1

As per 2016 estimates, approximately one in four deaths and one in five disability-adjusted life years (DALYs) lost were attributable to environmental stressors globally (1). Humans are exposed to environmental stressors through numerous sources for example unsafe food, water and sanitation, ambient air pollution, indoor air pollution from solid biofuels, irrational use of agrochemicals, inadequate disposal of domestic and industrial wastes, and global climate change. The impact of environmental stressors appears to vary strikingly across the lifespan and by gender and socio-economic status, making women and children in low- and middle-income countries (LMICs) disproportionately more vulnerable. The human health effects of environmental exposures are diverse as well. For instance, exposure to combustion products from fossil fuels may lead to adverse birth outcomes, developmental effects, cardiometabolic and respiratory diseases, and cancers.

Environmental pollution is embedded in several health- and environment-related SDG goals and targets. Despite this, there are still substantial knowledge gaps and a lack of awareness among stakeholders regarding the adverse health and nutritional effects of different environmental toxicants. The prevention and control of environmental toxicants are also not prioritized in any of the relevant strategies and policies, particularly in many LMICs. Many nutrients, including antioxidant vitamins and trace elements, can sometimes minimize or even counteract the detrimental effects of environmental toxicants. In this Research Topic titled "*Role of Toxicants, Pollutants, and Trace Elements in Health and Nutrition*,” there are five studies linking environmental stressors with population health and nutrition. This Research Topic includes original articles, systematic and narrative reviews, and an animal study. One study assessed the importance of adequate nutrition to prevent disease and four studies covered exposure to various environmental toxicants present in food and/or drinking water, one of these studies also covered toxicant-antioxidant interaction.

Evidence suggests that consuming healthy diets with high levels of anti-oxidants and anti-inflammatory properties can reduce vulnerability to non-communicable diseases (2, 3). Understanding the relationship between nutritional modulations of environmental toxicants and susceptibility to disease development is important for both cumulative risk assessment and for future public health interventions (2). Liu et al. evaluated associations between dietary trace element intake and erectile dysfunction (ED), a common clinical condition that affects men over the age of 40. The results suggested that increasing dietary intake of magnesium (Mg), zinc (Zn), copper (Cu), and selenium (Se) was associated with decreased ED prevalence (all *P* < 0.001). However, a relevant limitation of the study is the outcome variable, self-assessed erectile dysfunction without medical validation. Since the causes of ED include both biological as well as psychological disorders, it cannot be excluded that self-assessment might not be an indicator of ED-related medical problems but rather the result of psychological problems.

Arsenic exposure through drinking water and food affects millions of people across the world and has been linked to cancers and cardiometabolic diseases, including skin lesions like dermatitis. Interestingly, a deficiency of the antioxidant vitamin A has been linked to worsening atopic dermatitis. Qin et al. explored the therapeutic mechanisms of vitamin A in the treatment of arsenic-related dermatitis through integrated *in silico* approaches of network pharmacology and molecular docking. Their investigation indicates that vitamin A induces multiple immunoregulation-associated functions, including interferon-gamma production and negative regulation of T-cell activation, suggesting that vitamin A may be used for treating arsenic-related dermatitis. Future studies are needed to explore the therapeutic window of vitamin A supplementation to treat arsenic-related dermatitis.

Acrylamide is classified as a probable human carcinogen (class 2A) according to the International Agency for Research on Cancer (IARC). Exposure to acrylamide occurs primarily through diet, particularly when high carbohydrate food is cooked at high temperatures and low moisture. Filippini et al. performed a systematic review and dose-response meta-analysis of epidemiological studies in PubMed, Scopus, and Web of Science databases to evaluate the association between dietary acrylamide exposure and several site-specific cancers using a random-effects meta-analysis. Their pooled analysis demonstrated no association between the highest vs. lowest dietary acrylamide exposure and each site-specific cancer investigated (e.g., colorectal, prostate, renal, pancreatic, and esophageal), with no evidence of thresholds in the dose-response meta-analysis.

Climate changes, including heavy and long-duration rainfall and excessive humidity are likely to increase the presence of various mycotoxins in food. In an animal study published in this Research Topic, Subramaniam et al. have explored the mechanisms on how aflatoxin B1 affects the gut and brain health leading to depressive-like behavior, using a randomized controlled trial of 32 male Sprague Dawley rats. Their research demonstrated that aflatoxin B1 exposure leads to anhedonia-like behavior, prolonged immobility time, and altered fecal bacterial profile among the exposed rats compared to the control rats.

Multiple independent studies have identified inorganic contaminants, including heavy metals, in foods designed for infants and young children. The narrative review by Bair has evaluated the current policies to safeguard against excessive concentrations of heavy metals in foods for infants and toddlers in the US. The review suggests that there is a lack of authoritative regulatory reference limits for heavy metals in infants' and children's food. Food manufacturers often self-determine their own internal standards for toxic heavy metals of their products.

In summary, the results of the above-mentioned studies highlight the importance of maintaining adequate nutrition to improve public health, and to reduce and sometimes perhaps even counteract toxicant-related diseases. However, foremost, the results demonstrate that it is essential to reduce the exposure to various environmental toxicants *via* food, drinking water, and air as much as possible to improve global health and that there is a lot of work to be done with implementing policies to make this a reality. The variety of environmental toxicants also stresses the importance of increasing the knowledge of mixtures of environmental toxicants, interactions with nutrition and the impact on health.

## Author contributions

SR wrote the introduction. SR and AN wrote the central part with comments to the cited papers and references. MK wrote the summary. All authors contributed to the article and approved the submitted version.
